# Identification and Association of Single Nucleotide Polymorphisms of the FTO Gene with Indicators of Overweight and Obesity in a Young Mexican Population

**DOI:** 10.3390/genes14010159

**Published:** 2023-01-06

**Authors:** Alonso Chama-Avilés, Karla Lucero Flores-Viveros, Jorge Alberto Cabrera-Ayala, Adriana Aguilar-Galarza, Willebaldo García-Muñoz, Lorenza Haddad-Talancón, Ma. de Lourdes Anzures-Cortés, Claudia Velázquez-Sánchez, Jorge Luis Chávez-Servín, Miriam Aracely Anaya-Loyola, Teresa García-Gasca, Víctor Manuel Rodríguez-García, Ulisses Moreno-Celis

**Affiliations:** 1 Facultad de Ciencias Naturales, Universidad Autónoma de Querétaro, Juriquilla, Querétaro 76230, Mexico; 2 Facultad de Enfermería, Universidad Autónoma de Querétaro, Las Campanas, Querétaro 76010, Mexico; 3 Facultad de Medicina, Unidad Saltillo, Universidad Autónoma de Coahuila, Coahuila 25000, Mexico; 4 Servicio Universitario de Salud, Secretaria de Atención a la Comunidad Universitaria, Universidad Autónoma de Querétaro, Las Campanas, Querétaro 76010, Mexico; 5 Laboratorio de Genética Humana, Código 46, S.A. de C.V., Cuernavaca 62498, Mexico; 6 Tecnologico de Monterrey, Escuela de Ingeniería y Ciencias, San Pablo, Querétaro 76130, Mexico

**Keywords:** *FTO* gene, obesity, single nucleotide polymorphisms

## Abstract

(1) Background: obesity is a global public health problem; various factors have been associated with this disease, and genetic factors play a very important role. Previous studies in multiple populations have associated a gene with fat mass and obesity (*FTO*). Thus, the present work aims to identify and determine associations between genetic variants of *FTO* with indicators of overweight and obesity in the Mexican population. (2) Methods: a total of 638 subjects were evaluated to compile data on body mass index (BMI), the percentage of body fat (%BF), the waist circumference (WC), the serum levels of triglycerides (TG), and food consumption. A total of 175 genetic variants in the *FTO* gene were sampled by a microarray in the evaluated population, followed by association statistical analyses and comparisons of means. (3) Results: a total of 34 genetic variants were associated with any of the 6 indicators of overweight and obesity, but only 15 showed mean differences using the recessive model after the Bonferroni correction. The present study shows a wide evaluation of *FTO* genetic variants associated with a classic indicator of overweight and obesity, which highlights the importance of genetic analyses in the study of obesity.

## 1. Introduction

Obesity is a global health problem [1]. Studies in various populations have shown the importance of the genetic component in obesity. Studies in twins have revealed that 80% of variations in the body mass index (BMI) are related to a genetic component [2]; other studies have reported that adopted children have more BMI alterations compared to those shown by their biological parents, indicating that 63% of these alterations resulted from a hereditary component and 31% from environmental factors. Furthermore, studies based on single nucleotide polymorphisms (SNP) have only been able to attribute 3% of BMI variation to a genetic effect [3,4]. Available data suggest a susceptibility of some populations to have higher figures of obesity, as in the case of the Latin American population, where obesity statistics have increased alarmingly and Mexico is considered the country with the highest rate of obesity. Both adult and child populations [5,6] are susceptible to being diagnosed as obese in the same proportion. Several independent studies have shown an association between *FTO* SNPs and fat mass and obesity [7,8,9].

*FTO* has been studied for several years and is known to encode for an enzyme nucleic acid methylase, dependent on α-ketoglutarate and iron (Fe II), which is ubiquitous in human tissues. Understanding the exact mechanism by which it is associated with obesity has been difficult [10,11]. However, a loss of function in homozygous *FTO* carriers has been observed to cause growth retardation and central nervous system disorders. Likewise, there are thin and obese heterozygous individuals, which indicates that the loss or gain of the *FTO* function is not a condition for the development of obesity but rather specific modifications in their activity. Such modifications can be due to subtle changes in the gene sequence, such as SNPs [11].

In a study investigating adipocytes derived from human adipose tissue, the researchers observed that the presence of the risk allele of SNP rs1421085 promoted a greater darkening of fat cells [12]. On the other hand, in studies looking directly at humans, the association of various SNPs has been observed in different populations in recent decades. Such is the case for SNPs rs9939609, rs6499640, rs8050136, and rs1558902 in the Chinese population [13]. A positive association was also observed with high BMI in the Korean population for SNPs rs1421085 and rs17817449 [13]. Furthermore, rs1421085, rs17817449, and rs9939609 have been associated with obesity in European populations, while these relationships differed in Melanesian, Micronesian, and Polynesian populations [14].

In the Mexican population, some associations of *FTO* SNPs with obesity of the SNPs rs1121980, rs17817449, rs3751812, and rs9930506 have been observed in the mestizo population [7], and rs9939609 and rs1421085 were associated with obesity in the Mayan population [15].

As these previous studies have not been able to analyze a greater number of genetic variants associated with phenotypic obesity markers, the present work focuses on the evaluation of 175 *FTO* SNPs filtered from a microarray to investigate their potential associations with common indicators of obesity phenotypes.

## 2. Materials and Methods

### 2.1. Subjects and Genetic Sampling

A total of 638 subjects were included in this study from the SUSALUD-UAQ, an initiative that seeks to determine the risk factors of the main non-communicable diseases in the young population. Participants who met the following criteria were included: men and women with an age range of 18 to 22 years, who agreed to sign the informed consent letter and who had a complete evaluation. Likewise, those with previously diagnosed chronic diseases such as cancer, diabetes, cardiovascular disease, women with polycystic ovarian syndrome, pregnant or lactating women, and those who had thyroid problems were excluded from the study; those who did not have complete evaluation information were eliminated.

From this evaluation, the anthropometric parameters of height (m) and weight (Kg) were selected for the calculation of the BMI (kg/m^2^) and waist circumference (cm) in the same way as the percentage of body fat, determined by 4-pole multifrequency bioelectrical impedance, using the mBCA Mod. 514 equipment (SECA, Hamburg, Germany). Likewise, biochemical parameters of glucose, TG, cholesterol, and HDL were determined from a blood sample extracted by venipuncture, enzymatic methods (SPINREACT, Girona, Spain), and using the Chemistry Analyzer Mod. BS 120 automated equipment (Mindray, Shenzhen, China).

Since there is no accurate diagnosis of obesity, this study took as markers of obesity those that have been found to be the best predictors of obesity and its comorbidities: body mass index (BMI), waist circumference (WC), and body fat percentage (BF%) [16], as well as elevated triglyceride levels [17] and high energy intake [18]. The following values were used as obesity parameters: body mass index > 25.0 kg/m^2^; WC in women > 80 cm and in men > 90 cm; percentage of body fat in women > 35% and in men > 20%; TG > 150 mg/dL; and energy intake > population median (>2400 Kcal). Fasting glucose < 100 mg/dL was used to rule out diabetes mellitus.

### 2.2. Analysis of Genetic Material

Subjects’ DNA samples were obtained from whole blood, using the QIAamp 96 DNA blood kit (QIAGEN, Illumina, CA, USA) and following the supplier’s specifications. The Illumina Infinium HTS Automated protocol, along with the Beadchip Global Screening Array microarray (GSA-24 v1.0), were used for human genotyping [19,20] in the Código 46 Genetics Laboratories. Data from 216 genetic variants on the *FTO* gene were initially recovered from the whole 669,672 variants on the Illumina microarray. We applied two data filters using PLINK, the percentage of missing variants per sample below 0.05, and the quality per individual with a call rate above 0.95 [21], which resulted in 175 variants on *FTO* fulfilling these filters. Genotypic and allelic frequencies were determined with GenAlEx 6.51 [22]. Null alleles were excluded from the dataset prior to further analyses; all markers were analyzed for the Hardy–Weinberg equilibrium (HWE) (Table S1).

### 2.3. Statistical Analysis

Statistical and descriptive analyses were performed to determine the general characteristics of the population. For the present study, the genotypes were grouped according to the additive model. For the recessive model, the alternative homozygous (xx) and reference homozygous plus heterozygous (XX + Xx) models were used, while for the dominant model, the reference homozygote (XX) and the set of heterozygote and alternative homozygotes were used. Binary logistic regressions were performed to determine significant associations (*p* ≤ 0.05) between genetic variants and indicators of obesity. Student’s *t*-tests (*p* ≤ 0.05) were performed to compare the means of the indicators of obesity for each of the models. One-way ANOVAs were performed to describe the mean differences between the homozygous reference, heterozygous, and homozygous risk populations, followed by the Bonferroni adjustment (*p* ≤ 0.05). All statistical analyses were performed using the Statistical Package for the Social Sciences (IBM SPSS Statistics for Macintosh, Version 23.0., Armonk, NY, USA: IBM Corp) [23].

## 3. Results

### 3.1. Description of the Population

From the studied population, 307 were men (48.27%) and 329 were women (51.73%); the mean age of the population was 19.34 years: 19.58 years for men and 19.12 years for women. Data from the variables related to obesity showed that the mean WC for men was 84.03 ± 11.46 cm and for women 77.88 ± 11.53 cm; the mean BMI for men was 24.03 ± 4.09 Kg/m^2^, and for women it was 23.41 ± 4.38 Kg/m^2^. The BF% data presented a mean of 21.13 ± 7.94% in men and 31.21 ± 7.31% in women; the average value of TG in serum was 114.34 ± 72.45 mg/dL in men and 96.67 ± 9.16 mg/dL in women; the calorie consumption values for men were 2584.6 ± 952.97 Kcal/day and 2227.6 ± 815.60 Kcal/day for women (Table 1).

**Figure 1:**
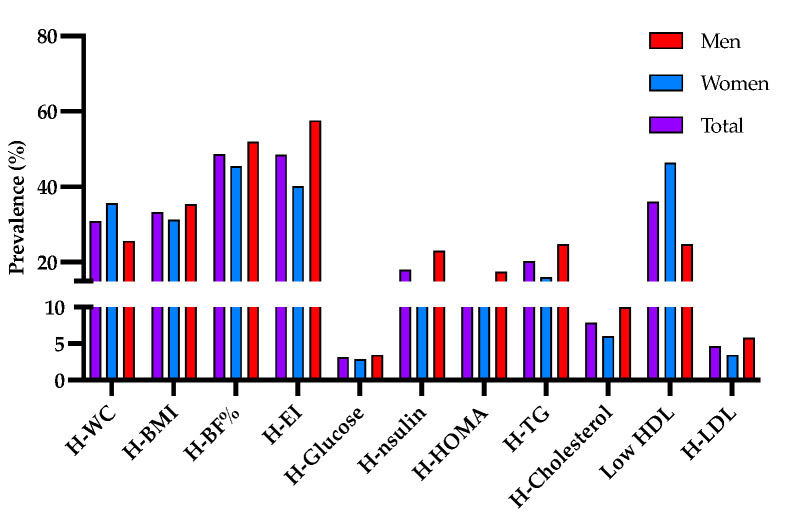
Prevalence of clinical markers associated with indicators of overweight and obesity. Total prevalence (purple bar); of women (blue bar) and men (red bar) with high waist circumference (H-WC); body mass index greater than 25 kg/m^2^ (H-BMI); high body fat percentage (H-BF%); energy intake greater than 2400 Kcal/day (H-EI); hyperglycemia (H-Glucose); hyperinsulinemia (H-Insulin); elevated HOMA index (H-HOMA); hypertriglyceridemia (H-TG); hypercholesterolemia (H-Cholesterol); low levels of HDL (Low-HDL); and elevated levels of LDL (H-LDL).

Likewise, a prevalence of high waist circumference was observed in the population of 30.8%, being 25.6% for men and 35.6% for women; according to the BMI > 25 Kg/m^2^, 33.23% of the population was overweight or obese, being higher in men than in women. On the other hand, the percentage of high body fat was shown in 48.55% of the population, and the energy consumption was greater than the population average (2400 Kcal/day) was observed high in 57.5% of men and 40.18% of women. Hyperglycemia was observed in 3.1% of the population, while hypercholesterolemia was observed in 7.89%. Hypertriglyceridemia (20.3%) and low HDL (36.02) were the most prevalent biochemical markers in the studied population (Figure 1).

### 3.2. Associations of FTO SNPs with Indicators of Obesity

According to the used model (xx − (Xx + XX)), significant associations (*p* = 0.05) between 34 genetic variants were associated with the risk (OR > 1) to 6 obesity indicators (WC, BMI > 25 Kg/m^2^, BMI > 30 Kg/m^2^, BF%, TG, and energy intake > 2400 Kcal). The genetic variants rs17219983, rs1966435, and rs12051261 showed a protective effect (OR < 1) for BMI > 25 Kg/m^2^, as well as the rs2111650, rs1966435, and rs12051261 to the BF%. This was the same with rs3751813 for serum TG, but energy consumption (>2400 Kcal) was negatively associated with the SNPs rs1075440 and rs7191566. Significant risk associations (OR > 1) were also observed between high WC and rs17817964 and rs6499662. Similarly, significant risk associations were found for between BMI > 25 Kg/m^2^ and SNPs rs8043785, rs35510800, rs6499662, and rs12931859. On the contrary, BMI > 30 Kg/m^2^ was positively associated with the genetic variants rs16945088, rs17817449, rs8043757, rs12931859, and rs7194243, and rs4389136, rs8043785, rs12232391, rs7194243, and energy consumption greater than 2400 Kcal/Day were associated with high BF%, as well as rs9939973, rs9940128, rs1421085, rs3751812, rs9936385, rs11075990, rs9939609, rs7202116, rs7185735, rs9941349, rs17817964, rs9922619, rs12149832, rs12149832, rs12149832, and rs9929152 (Table 2).

On the other hand, according to the analysis of the dominant model (XX − (Xx + xx)), rs1421091, rs4389136, rs12232391, rs7200972, rs12931859, and rs7194243 resulted in significant associations for risk at a high percentage of fat, while rs10852523, rs61743972, rs3826169, and rs7203572 were associated with obesity (BMI >30 Kg/m^2^) and rs4389136 with hypertriglyceridemia. Protective effects were also observed of rs7205986, rs7203521, rs6499640, rs2111650, rs17819033, rs17219983, rs9934504, rs56335873, rs12933996, rs35090620, and rs16952686 for high fat percentage, and rs9934504, rs12933996, rs16952686, and rs1966435 for BMI > 25 Kg/m^2^; while rs9934504 turned out to be a protective factor for high waist circumference, and rs74018195, rs74449711, rs7191566, rs17820328, and rs16952657 for calorie intake greater than the median of the studied population (Table 3).

### 3.3. Comparison of Means BMI, %BF, WC, TG, and Energy Consumption with Additive Models and Genotype

Mean comparisons by Student’s *t*-test showed statistically significant differences (*p* ≤ 0.05) only among WC, BMI, and BF% and 16 genetic variants. Specifically, WC with rs12232391; BMI with rs12232391 and rs12051261; and percentage of body fat with rs12232391 and rs12051261; rs9939973, rs9940128, rs1421085, rs17817449, rs3751812, rs9936385, rs11075990, rs9939609, rs7206629, rs7202116, rs7185735, rs9941349, rs17817964, and rs12051261 (Table 4).

According to the results of Student’s *t*-test for the dominant model (XX − (Xx + xx)), differences can be observed in the means of waist circumference in the SNPs rs1421091, rs4389136, and rs16952686, as well as in the BMI means in the variants rs9934504, rs56335873, rs12232391, and rs16952686. Likewise, significant statistical differences for the percentage of fat were observed in rs7191566, rs1421085, rs3751812, rs17817964, rs2111650, rs11642841, rs9934504, rs56335873, rs12232391, and rs7200972; in the same way as for triglyceride levels, the variants rs17817449, rs8043757, rs9936385, rs11075990, rs9939609, rs7202116, and rs7185735 affected the means of the studied population; likewise, the average energy consumption was affected in this model by the variants rs74449711, rs16952657, and rs35510800 (Table 5).

The results showed significant statistical differences (*p* ≤ 0.05, ANOVA) between the means of 12 of the determined genetic variants for WC of the SNPs rs12232391 and rs17817449, while for BMI, the variants that showed differences were rs17817449 and rs12232391. For the percentage of body fat, the differences were observed with rs7191566, rs1421085, rs17817449, rs3751812, rs17817964, rs2111650, and rs12232391. On the other hand, energy consumption also showed significant statistical differences (*p* ≤ 0.05) in the variants rs9936385, rs11075990, rs9939609, rs7202116, and rs7185735 (Table 6).

It is important to mention that only four variants passed the Bonferroni adjustment: rs12232391 for WC, rs17817449 and rs12232391 for BMI, and rs17817449, rs3751812, rs2111650, and rs12232391 for %BF. None of the variants associated with energy consumption passed the Bonferroni adjustment (Table 6, highlighted in bold).

## 4. Discussion

Recent studies have shown the importance of the *FTO* gene in the development of the organism. Studies in experimental mice showed that suppression of *FTO* leads to reduced body weight and body mass, while overexpression promotes an increase in body mass, fat mass, and food consumption [24]. Therefore, *FTO* and downstream genes regulated from non-coding regions, mainly IRX3 and IRX5, which are genes related to neural development in areas associated with food consumption and may be valuable therapeutic targets for obesity [25,26] The effects of SNPs have been observed to be differential between populations; such is the case of rs9930506, which was observed to have risk associations with BMI in the European population but not in the Asian population [27].

The present work analyzed 175 genetic variants of *FTO*, of which only 34 were associated with any of the indicators of overweight and obesity, while only 16 of these variants showed differences in means according to the recessive model of the minor allele and 12 differences in the average of the indicators of overweight and obesity according to the genotype present, whereas only 4 passed the Bonferroni adjustment. From these last variants, rs12232391 showed differences between the population means in WC, BMI, and BF%; however, it has not been reported in association with any condition.

The variant rs17817449 has been extensively studied, and its effects have been observed in different populations, such as in the case of a study in an Iranian population with type 2 diabetes mellitus [28] and obesity [29]. This has also been replicated in Chinese women [30]. In the current study, the association of this marker with BMI >30 Kg/m^2^ and with higher energy consumption was observed, while differences were observed in the means of WC, BMI, and percentage of body fat. The genetic variant rs7191566 was observed in a population study in Mexico but not included in further analyses because it was not in Hardy-Weinberg equilibrium [7]. Interestingly, in the current work, this marker showed a different genotype when compared with the mean percentage of fat in the population.

In our results, variant rs1421085 was found to be associated with high consumption of Kcal, and mean differences were observed in the percentage of body fat. This marker has been extensively studied, and recent studies have found higher allelic frequencies in people with obesity as well as its associations with higher triglyceride and cholesterol levels in Turkish children [31]. Likewise, rs1421085 was previously reported with higher allelic frequencies in the Iranian population, showing its association with obesity markers [32]. In the adult Mexican obese population, rs1421085 has also been considered a genetic marker of risk [15]. On the other hand, rs3751812 has been associated with obesity in the Taiwanese population, as well as the reduction of its effects by increasing the physical activity for this population [33], while in a population of Greek adults, when analyzing the same SNP, it was not found in Hardy–Weinberg equilibrium, explaining why it had to be discarded from the study [34]. Similarly, in a study in the Polish population, it was observed that people carrying this polymorphism tended to have higher levels in the blood lipid profile [35].

Our findings show a positive association between energy consumption greater than 2400 Kcal and the said marker, in addition to differences in the means of body fat, both in the additive model and in the complete genotype. Similarly, rs17817964 in this study was associated with WC and energy consumption greater than the population median. It should be noted that this genetic marker was also associated with obesity in African-American women over 18 years of age with low birth weight [36], and it was generally observed as associated with obesity in people with African-American ancestry [37]. The variant rs2111650 was associated with the percentage of body fat and showed differences in the means of %BF related to its polymorphism; however, it has not been identified as a risk or protective variant in any other population. Furthermore, rs2111650 has been associated in various genome-wide association studies, mainly with obesity markers [38,39].

The rs9936385, rs7202116, and rs11075990 did not pass the Bonferroni adjustment and associations with obesity markers have not been observed in other studies. Interestingly, rs9939609 is one of the variants of the *FTO* gene that has been most researched for its relationship with clinical obesity markers in various populations, including the Mexican child and adult populations [40,41]. It was also associated with hyperglycemia in women from southern Mexico with metabolic syndrome [42]. In the current study, rs9939609 showed mean differences in the percentage of body fat according to the recessive model and with a higher energy intake, without exceeding the Bonferroni adjustment.

Finally, although *FTO* is a possible genetic marker for obesity as we have discussed throughout the previous lines when comparing the results of various studies in different populations, further population studies are required to corroborate it as a genetic marker for obesity risk.

## 5. Conclusions

Some genetic variants of *FTO* showed a strong relationship with indicators of obesity in the studied population, opening the possibility for specific studies on a population previously diagnosed as obese to confirm the specific influence of the genetic variants identified in this study, since the results obtained were carried out in a young population of mestizo Mexicans without diagnosed diseases.

## Figures and Tables

**Table 1:** General characteristics of the population.

	Men (307)		Women (329)		Total	
	Mean	S.D.	Mean	S.D.	Mean	S.D.
Age (years)	19.58	3.46	19.12	1.94	19.34	2.78
Weight (Kg)	70.75	13.15	59.55	11.88	1.653	8.95
Height (m)	1.715	7.03	1.595	6.24	64.95	13.7
Waist circumference (cm)	84.03	11.46	77.88	11.53	80.85	11.89
BMI(Kg/m^2^)	24.03	4.09	23.41	4.38	23.71	4.25
Body fat (%)	21.13	7.94	31.21	7.31	26.35	9.13
Energy intake (Kcal)	2584.6	952.97	2227.6	815.60	2406.1	902.51
Glucose (mg/dL)	85.03	8.97	82.44	9.16	83.68	9.16
Insulin (mg/dL)	7.71	5.12	7.92	6.03	7.82	5.62
HOMA-Index	1.62	1.14	1.61	1.23	1.62	1.94
Triglycerides (mg/dL)	114.34	72.45	96.67	9.16	105.14	63.79
Total cholesterol (mg/dL)	157.57	32.65	157.88	27.76	157.74	30.17
LDL (mg/dL)	86.62	24.96	85.41	22.66	85.79	23.78
HLD (mg/dL)	48.053	11.02	53.27	13.28	50.76	12.51

BMI—Body mass index; HOMA—Homeostatic model assessment; LDL—Low density lipoprotein; and HDL—High density lipoprotein.

**Table 2:** Association of *FTO* SNPs with indicators of overweight and obesity, recessive model.

Clinical Marker	SNP	Model	OR	CI95%		*p*-Value	Effect
Hight waist circumference (cm)	rs17817964	CC + CT	2.387	1.056	5.394	0.037	Risk
		TT *					
	rs6499662	AA + AG	2.412	1.051	5.537	0.038	Risk
		GG *					
BMI > 25 Kg/m^2^	rs8043785	AA + AG	1.863	1.078	3.219	0.026	Risk
		GG *					
	rs35510800	GG + GA	1.920	1.070	3.444	0.029	Risk
		AA *					
	rs6499662	AA + AG	2.514	1.101	5.737	0.029	Risk
		GG *					
	rs12931859	CC + CT	1.644	1.057	2.559	0.028	Risk
		TT *					
	rs17219983	CC + CT	0.477	0.231	0.986	0.046	Protective
		TT *					
	rs1966435	TT + TC	0.641	0.418	0.981	0.041	Protective
		CC *					
	rs12051261	CC + CT	0.475	0.246	0.917	0.026	Protective
		TT *					
BMI > 30 Kg/m^2^	rs16945088	AA + AG	3.535	1.253	9.974	0.017	Risk
		GG *					
	rs17817449	TT + TG	2.414	1.154	5.050	0.019	Risk
		GG *					
	rs8043757	AA + AT	2.167	1.042	4.505	0.038	Risk
		TT *					
	rs12931859	CC + CT	2.181	1.104	4.310	0.025	Risk
		TT *					
	rs7194243	CC + CT	1.960	1.062	3.618	0.031	Risk
		TT *					
Hight body fat (%)	rs4389136	AA + AG	1.616	1.098	2.38	0.015	Risk
		GG *					
	rs8043785	AA + AG	1.902	1.091	3.317	0.023	Risk
		GG *					
	rs12232391	TT + TG	1.436	1.006	2.050	0.046	Risk
		GG *					
	rs7194243	CC + CT	1.518	1.054	2.186	0.025	Risk
		TT *					
	rs2111650	TT + TC	0.307	0.095	0.992	0.048	Protective
		CC *					
	rs1966435	TT + TC	0.635	0.431	0.936	0.022	Protective
		CC *					
	rs12051261	CC + CT	0.534	0.306	0.932	0.027	Protective
		TT *					
Hight triglycerides (mg/dL)	rs3751813	GG + GT	0.477	0.238	0.957	0.037	Protective
		TT *					
Energy intake > 2400 Kcal	rs9939973	GG + GA	1.866	1.000	3.484	0.050	Risk
		AA *					
	rs9940128	GG + GA	1.866	1.000	3.484	0.050	Risk
		AA *					
	rs1421085	TT + TC	1.966	1.005	3.842	0.048	Risk
		CC *					
	rs3751812	GG + GT	2.772	1.112	6.912	0.029	Risk
		TT *					
	rs9936385	TT + TC	2.738	1.160	6.462	0.022	Risk
		CC *					
	rs11075990	AA + AG	2.738	1.160	6.462	0.022	Risk
		GG *					
	rs9939609	TT + TA	2.738	1.160	6.462	0.022	Risk
		AA *					
	rs7206629	TT + TC	1.866	0.999	3.482	0.050	Risk
		CC *					
	rs7202116	AA + AG	2.738	1.160	6.462	0.022	Risk
		GG *					
	rs7185735	AA + AG	2.738	1.160	6.462	0.022	Risk
		GG *					
	rs9941349	CC + CT	2.213	1.121	4.366	0.022	Risk
		TT *					
	rs17817964	CC + CT	2.560	1.018	6.439	0.046	Risk
		TT *					
	rs9922619	GG + GT	2.102	1.086	4.072	0.028	Risk
		TT *					
	rs12149832	GG + GA	2.427	1.022	5.765	0.045	Risk
		AA *					
	rs11642841	CC + CA	3.330	1.185	9.363	0.023	Risk
		AA *					
	rs9929152	GG + GA	1.444	1.019	2.048	0.039	Risk
		AA *					
	rs1075440	GG + GA	0.631	0.404	0.984	0.042	Protective
		AA *					
	rs7191566	AA + AG	0.243	0.067	0.879	0.031	Protective
		GG *					

BMI—Body mass index; OR—Odds ratio; CI95%—Confidence interval; (*) indicates the genotype considered at risk in the analysis; and *p*-value = 0.05.

**Table 3:** Association of *FTO* SNPs with indicators of overweight and obesity, dominant model.

Clinical Marker	SNP	Model	OR	CI95%		*p*-Value	Effect
Hight body fat (%)	rs1421091	AA	1.683	1.158	2.444	0.006	Risk
		CC + AC *					
	rs4389136	AA	1.616	1.125	2.321	0.009	Protective
		GG + AG *					
	rs12232391	TT	1.579	1.071	2.327	0.021	Risk
		GG + TG *					
	rs7200972	GG	2.094	1.256	3.491	0.005	Risk
		AA + GA *					
	rs12931859	CC	1.426	1.022	1.991	0.037	Risk
		TT + CT *					
	rs7194243	CC	1.486	1.037	2.128	0.031	Risk
		TT + CT *					
	rs7205986	AA	0.672	0.479	0.943	0.021	Protective
		GG + AG *					
	rs7203521	GG	0.698	0.506	0.963	0.028	Protective
		AA + GA *					
	rs6499640	GG	0.698	0.506	0.963	0.028	Protective
		AA + GA *					
	rs2111650	TT	0.655	0.448	0.958	0.029	Protective
		CC + TC *					
	rs17819033	GG	0.576	0.389	0.853	0.006	Protective
		TT + GT *					
	rs17219983	CC	0.615	0.445	0.849	0.003	Protective
		TT + CT *					
	rs9934504	GG	0.560	0.359	0.873	0.011	Protective
		AA + GA *					
	rs56335873	TT	0.590	0.379	0.917	0.019	Protective
		AA + TA *					
	rs12933996	GG	0.681	0.473	0.980	0.038	Protective
		AA + GA *					
	rs35090620	TT	0.603	0.422	0.861	0.005	Protective
		CC + TC *					
	rs16952686	AA	0.466	0.236	0.921	0.028	Protective
		GG + AG *					
Hight waist circumference (cm)	rs9934504	GG	0.590	0.357	0.975	0.040	Protective
		AA + GA *					
Energy intake > 2400 Kcal	rs35510800	GG	1.530	1.099	2.130	0.012	Risk
		AA + GA *					
	rs71392011	CC	4.114	1.120	15.104	0.033	Risk
		AA + CA *					
	rs74018195	TT	0.113	0.014	0.916	0.041	Protective
		CC + TC *					
	rs74449711	TT	0.080	0.010	0.631	0.017	Protective
		GG + TG *					
	rs7191566	AA	0.693	0.486	0.988	0.042	Protective
		GG + AG *					
	rs17820328	AA	0.414	0.176	0.971	0.043	Protective
		GG + AG *					
	rs16952657	CC	0.587	0.349	0.987	0.045	Protective
		TT + CT *					
BMI > 25 Kg/m^2^	rs9934504	GG	0.577	0.352	0.947	0.030	Protective
		AA + GA *					
	rs12933996	GG	0.686	0.472	0.999	0.049	Protective
		AA + GA *					
	rs16952686	AA	0.379	0.164	0.875	0.023	Protective
		GG + AG *					
	rs1966435	TT	0.685	0.481	0.975	0.036	Protective
		CC + CT *					
BMI > 30 Kg/m^2^	rs10852523	TT	2.998	1.420	6.328	0.004	Risk
		CC + CT *					
	rs61743972	GG	2.395	1.051	5.458	0.038	Risk
		CC + GC *					
	rs3826169	GG	4.459	1.062	18.725	0.041	Risk
		AA + GA *					
	rs7203572	AA	1.898	1.042	3.456	0.036	Risk
		CC + AC *					
Hight triglycerides (mg/dL)	rs4389136	AA	1.692	1.033	2.770	0.037	Risk
		GG + AG *					

BMI—Body mass index; OR—Odds ratio; CI95%—Confidence interval; (*) indicates the genotype considered at risk in the analysis; and *p*-value = 0.05.

**Table 4:** Comparison of indicators of overweight and obesity means in recessive model.

Clinical Marker	SNP	Model	Mean	S.D.	*p*-Value
Waist circumference (cm)	rs12232391	(TT + TG)	80.35	11.9	0.043
		GG	82.47	11.78	
BMI Kg/m^2^	rs12232391	(TT + TG)	23.47	4.21	0.024
		GG	24.33	4.41	
	rs12051261	(CC + TC)	23.87	4.31	0.012
		TT	22.42	3.86	
Body fat (%)	rs9939973	(GG + GA)	26.19	9.26	0.027
		AA	28.79	7.46	
	rs9940128	(GG + GA)	26.19	9.26	0.027
		AA	28.79	7.46	
	rs1421085	(TT + TC)	26.18	9.23	0.033
		CC	29.29	7.63	
	rs17817449	(TT + TG)	26.02	9.14	0.007
		GG	29.07	8.87	
	rs3751812	(GG + GT)	26.21	9.17	0.018
		TT	30.63	7.8	
	rs9936385	(TT + TC)	26.22	9.19	0.038
		CC	29.9	7.73	
	rs11075990	(AA + AG)	26.22	9.19	0.038
		GG	29.9	7.73	
	rs9939609	(TT+ TA)	26.22	9.19	0.038
		AA	29.9	7.73	
	rs7206629	(TT + TC)	26.17	9.26	0.040
		CC	28.99	7.5	
	rs7202116	(AA + AG)	26.22	9.19	0.038
		GG	29.9	7.73	
	rs7185735	(AA + AG)	26.22	9.19	0.038
		GG	29.9	7.73	
	rs9941349	(CC + CT)	26.19	9.23	0.044
		TT	29.13	7.72	
	rs17817964	(CC + CT)	26.23	9.18	0.031
		TT	30.34	7.84	
	rs12051261	(CC + TC)	26.73	9.17	0.006
		TT	23.33	8.56	

BMI: body mass index; S.D. = Standard deviation; *p*-value = 0.05.

**Table 5:** Comparison of indicators of overweight and obesity means in dominant model.

Clinical Marker	SNP	Model	Mean	S.D.	*p*-Value
Waist circumference (cm)	rs1421091	AA	79.50	10.45	0.049
		(AC + CC)	81.46	12.33	
	rs4389136	AA	79.53	10.41	0.042
		(AG + GG)	81.51	12.40	
	rs16952686	AA	81.215	12.0515	0.011
		(AG + GG)	77.368	8.8911	
BMI Kg/m^2^	rs9934504	GG	23.90	4.34	0.022
		(GA + AA)	22.84	3.90	
	rs56335873	TT	23.87	4.34	0.046
		(TA + AA)	22.95	3.94	
	rs12232391	TT	22.99	3.77	0.021
		(TG + GG)	23.93	4.40	
	rs16952686	AA	23.818	4.3441	0.011
		(AG + GG)	22.438	3.1919	
Body fat (%)	rs7191566	AA	27.04	9.22	0.013
		(AG + GG)	25.09	8.92	
	rs1421085	TT	25.82	9.12	0.040
		(TC + CC)	27.40	9.16	
	rs3751812	GG	25.82	9.13	0.031
		(GT + TT)	27.50	9.13	
	rs17817964	CC	25.86	9.11	0.048
		(CT + TT)	27.41	9.19	
	rs2111650	TT	26.95	9.10	0.007
		(CT + CC)	24.64	9.14	
	rs11642841	CC	25.86	9.09	0.042
		(CA + AA)	27.46	9.22	
	rs9934504	GG	26.80	9.21	0.013
		(GA + AA)	24.32	8.64	
	rs56335873	TT	26.76	9.22	0.023
		(TA + AA)	24.49	8.62	
	rs12232391	TT	24.73	8.92	0.015
		(TG + GG)	26.88	9.18	
	rs7200972	GG	24.3212	8.87798	0.036
		(GA + AA)	26.6877	9.17234	
Triglycerides (mg/dL)	rs17817449	TT	107.58	70.10	0.028
		(TG + GG)	96.43	52.05	
	rs8043757	AA	107.68	70.17	0.025
		(AT + TT)	96.30	51.96	
	rs9936385	TT	107.67	70.17	0.025
		(TC + CC)	96.31	51.96	
	rs11075990	AA	107.67	70.17	0.025
		(AG + GG)	96.31	51.96	
	rs9939609	TT	107.67	70.17	0.025
		(TA + AA)	96.31	51.96	
	rs7202116	AA	107.67	70.17	0.025
		(AG + GG)	96.31	51.96	
	rs7185735	AA	107.67	70.17	0.025
		(AG + GG)	96.31	51.96	
Energy intake (Kcal/day)	rs74449711	TT	2518.26	906.91	0.001
		(TG + GG)	1731.67	404.54	
	rs16952657	CC	2534.22	914.66	0.020
		(CT + TT)	2265.71	808.14	
	rs35510800	GG	2430.1252	891.44287	0.049
		(GA + AA)	2577.7083	916.83851	

BMI—Body mass index; S.D.—Standard deviation; and *p*-value = 0.05.

**Table 6:** Association of the genotype with indicators of overweight and obesity.

Clinical Marker	SNP	Genotype	Mean	S.D.	*p*-Value	*p*. adj
Waist circumference (cm)	rs12232391	TT	79.32	11.76	0.058	**0.052**
		TG	80.84	11.96		
		GG	82.47	11.78		
	rs17817449	TT	81.51	11.94	0.042	0.061
		TG	78.89	10.54		
		GG	82.2	13.74		
BMI (Kg/m^2^)	rs17817449	TT	23.82	4.33	0.04	**0.047**
		TG	23.07	3.95		
		GG	24.52	4.54		
	rs12232391	TT	22.99	3.77	0.021	**0.016**
		TG	23.7	4.39		
		GG	24.33	4.41		
Body fat (%)	rs7191566	AA	27.04	9.22	0.027	0.089
		AG	25.29	8.96		
		GG	22.82	8.36		
	rs1421085	TT	25.82	9.12	0.04	0.059
		TC	26.96	9.45		
		CC	29.29	7.63		
	rs17817449	TT	26.12	9.08	0.026	**0.033**
		TG	25.78	9.32		
		GG	29.07	8.87		
	rs3751812	GG	25.82	9.13	0.019	**0.032**
		GT	27.08	9.24		
		TT	30.63	7.8		
	rs17817964	CC	25.86	9.11	0.035	0.059
		CT	27.02	9.3		
		TT	30.34	7.84		
	rs2111650	TT	26.95	9.1	0.027	**0.041**
		TC	24.73	9.32		
		CC	23.95	7.61		
	rs12232391	TT	24.73	8.92	0.027	**0.022**
		TG	26.5	9.28		
		GG	27.52	8.99		
Energy intake (Kcal/Day)	rs9936385	TT	2530.51	886.2	0.044	0.067
		TC	2399.05	897.45		
		CC	2831.74	1154.44		
	rs11075990	AA	2350.51	886.2	0.044	0.067
		AG	2399.05	897.45		
		GG	2831.74	1154.44		
	rs9939609	TT	2530.51	886.2	0.044	0.067
		TA	2399.05	897.45		
		AA	2831.74	1154.44		
	rs7202116	AA	2530.51	886.2	0.044	0.067
		AG	2399.05	897.45		
		GG	2831.74	1154.44		
	rs7185735	AA	2530.51	886.2	0.044	0.067
		AG	2399.05	897.45		
		GG	2831.74	1154.44		

BMI—Body mass index; S.D.—Standard deviation; *p*-value = 0.05; and *p*-adj. = *p*-value adjustment by Bonferroni correction. In bold type, the *p* values that were statistically significant in the Bonferroni correction

## Data Availability

The data presented in this study are available upon request from the corresponding author. The data is not publicly available as the overall project continues to analyze the results.

## Supplementary Materials

The following supporting information can be downloaded at: https://www.mdpi.com/article/10.3390/genes14010159/s1, Table S1: Genetic variants analyzed. Genotypes, reference and alternative alleles show their respective frequencies. HWE *p*-values are also included.
