# Supplementary material for: Identification and Association of Single Nucleotide Polymorphisms of the FTO Gene with Indicators of Overweight and Obesity in a Young Mexican Population
Source: Genes (Basel). 2023 Jan 6;14(1):159. doi: 10.3390/genes14010159 (PMC9858641; doi:10.3390/genes14010159)
Supplement: Supplementary file 1 [file genes-14-00159-s001.zip › genes-2045669-supplementary.pdf]

Table S1. Genetic variants analyzed. Genotypes, reference and alternative alleles show their respective frequencies. HWE p-values are also included.

SNP	Chromosome position	Reference/Alternative Alleles (Frequencies %)	Genotypes (Frequency %)	HWE p-value
**rs1421091**	53739773	A/C<br>(0.504/0.496)	AA (25.71)<br>AC (49.37)<br>CC (24.92)	0.753
**rs4389136**	53747858	A/G<br>(0.527/0.473)	AA (28.06)<br>AG (49.37)<br>GG (22.57)	0.809
**rs140352368**	53751902	G/T<br>(0.997/0.003)	GG (99.37)<br>GT (0.62)<br>TT (0)	0.937
**rs7205986**	53755146	A/G<br>(0.799/0.201)	AA (65.04)<br>AG (29.62)<br>GG (5.33)	0.046
**rs1421084**	53757740	A/G<br>(0.969/0.031)	AA (94.36)<br>AG (5.02)<br>GG (0.63)	0.000
**rs57976072**	53759099	C/T<br>(0.995/0.005)	CC (99.06)<br>CT (0.94)<br>TT (0)	0.905
**rs4386132**	53759123	C/T<br>(0.972/0.028)	CC (94.36)<br>CT (5.64)<br>TT (0)	0.463
**rs7202836**	53760532	G/A<br>(0.991/0.009)	GG (98.43)<br>GA (1.41)	0.000

			AA (0.16)	
**rs9925311**	53767648	G/A	GG (95.30)	0.298
		(0.976/0.024)	GA (4.55)	
			AA (0.15)	
**rs7203521**	53769293	G/A	GG (52.82)	0.732
		(0.725/0.275)	GA (39.34)	
			AA (7.84)	
**rs6499640**	53769677	G/A	GG (52.82)	0.732
		(0.725/0.275)	GA (39.34)	
			AA (7.84)	
**rs62048371**	53770081	A/G	AA (97.65)	0.004
		(0.987/0.013)	AG (2.19)	
			GG (0.15)	
**rs74018195**	53771105	T/C	TT (98.28)	0.826
		(0.991/0.009)	TC (1.72)	
			CC (0)	
**rs4396532**	53773047	G/A	GG (92.95)	0.846
		(0.946/0.036)	GA (6.90)	
			AA (0.15)	
**rs79977114**	53774354	A/G	AA (97.81)	0.779
		(0.989/0.011)	AG (2.19)	
			GG (0)	
**rs74449711**	53784964	T/G	TT (97.65)	0.004
		(0.987/0.013)	TG (2.19)	
			GG (0.15)	
**rs1861868**	53790402	C/T	CC (48.28)	0.740
		(0.693/0.307)	CT (42.00)	
			TT (9.72)	
**rs1075440**	53790906	G/A	GG (36.05)	0.322

		(0.592/0.408)	GA (46.40)<br>AA (17.55)	
**rs7191566**	53793204	A/G<br>(0816/0.184)	AA (65.99)<br>AG (31.19)<br>GG (2.82)	0.338
**rs13334933**	53795636	A/G<br>(0.897/0.103)	AA (81.03)<br>AG (17.40)<br>GG (1.57)	0.159
**rs6499642**	53797506	C/T<br>(0.978/0.022)	CC (95.92)<br>CT (3.76)<br>TT (0.31)	0.002
**rs8047395**	53798523	G/A<br>(0.574/0.426)	GG (36.68)<br>GA (41.38)<br>AA (21.94)	0.000
**rs9939973**	53800568	G/A<br>(0.731/0.269)	GG (54.08)<br>GA (38.08)<br>AA (7.84)	0.432
**rs9940128**	53800754	G/A<br>(0.731/0.269)	GG (54.08)<br>GA (38.09)<br>AA (7.83)	0.432
**rs1421085**	53800954	T/C<br>(0.787/0.213)	TT (64.26)<br>TC (28.84)<br>CC (6.89)	0.000
**rs16952520**	53803038	A/G<br>(0.861/0.140)	AA (74.45)<br>AG (23.20)<br>GG (2.35)	0.394
**rs1558902**	53803574	T/A<br>(0.778/0.222)	TT (60.19)<br>TA (35.26)<br>AA (4.54)	0.585

**rs55872725**	53809123	C/T (0.793/0.207)	CC (63.32) CT (31.97) TT (4.70)	0.516
**rs1121980**	53809247	G/A (0.723/0.277)	GG (52.98) GA (38.71) AA (8.30)	0.409
**rs73612011**	53809861	A/G (0.965/0.035)	AA (93.41) AG (6.11) GG (0.48)	0.010
**rs72803680**	53810635	A/G (0.882/0.118)	AA (78.36) AG (19.74) GG (1.88)	0.224
**rs62033399**	53810943	C/T (0.794/0.206)	CC (63.79) CT (31.19) TT (5.02)	0.236
**rs16945088**	53812524	A/G (0.886/0.114)	AA (81.19) AG (14.89) GG (3.91)	0.000
**rs17817449**	53813367	T/G (0.757/0.243)	TT (63.63) TG (24.13) GG (12.23)	0.000
**rs8043757**	53813450	A/T (0.751/0.249)	AA (63.32) AT (23.51) TT (13.17)	0.000
**rs11075987**	53815161	T/G (0.571/0.429)	TT (33.86) TG (46.55) GG (19.59)	0.210
**rs8050136**	53816275	C/A (0.780/0.220)	CC (63.48) CA (28.99)	0.000

			AA (7.52)	
**rs3751812**	53818460	G/T	GG (66.30)	0.402
		(0.811/0.189)	GT (29.62)	
			TT (4.08)	
**rs3751813**	53818708	G/T	GG (44.04)	0.001
		(0.641/0.359)	GT (40.13)	
			TT (15.83)	
**rs9936385**	53819169	T/C	TT (63.32)	0.646
		(0.794/0.206)	TC (32.13)	
			CC (4.55)	
**rs11075990**	53819893	A/G	AA (63.32)	0.646
		(0.794/0.206)	AG (32.13)	
			GG (4.55)	
**rs9939609**	53820527	T/A	TT (63.32)	0.646
		(0.794/0.206)	TA (32.13)	
			AA (4.55)	
**rs7206629**	53821413	T/C	TT (56.27)	0.117
		(0.742/0.258)	TC (35.89)	
			CC (7.83)	
**rs7202116**	53821615	A/G	AA (63.32)	0.646
		(0.794/0.206)	AG (32.13)	
			GG (4.55)	
**rs7185735**	53822651	A/G	AA (63.32)	0.646
		(0.794/0.206)	AG (32.13)	
			GG (4.55)	
**rs9941349**	53825488	C/T	CC (62.54)	0.004
		(0.778/0.222)	CT (30.56)	
			TT (6.89)	
**rs17817964**	53828066	C/T	CC (65.99)	0.597

		(0.810/0.190)	CT (30.09)	
			TT (3.91)	
**rs9930506**	53830465	A/G	AA (61.76)	0.000
		(0.640/0.361)	AG (4.39)	
			GG (33.86)	
**rs9922619**	53831771	G/T	GG (61.91)	0.003
		(0.774/0.226)	GT (30.88)	
			TT (7.21)	
**rs2111650**	53832816	T/C	TT (75.86)	0.109
		(0.867/0.133)	TC (21.63)	
			CC (2.51)	
**rs8044769**	53839135	T/C	TT (33.07)	0.000
		(0.495/0.505)	TC (32.91)	
			CC (34.01)	
**rs111357538**	53842712	G/A	GG (97.02)	0.703
		(0.985/0.015)	GA (2.97)	
			AA (0)	
**rs12149832**	53842908	G/A	GG (64.89)	0.436
		(0.803/0.197)	GA (30.72)	
			AA (4.39)	
**rs201041270**	53844134	G/A	GG (100)	-
		(100/0)	GA (0)	
			AA (0)	
**rs75547181**	53845040	A/G	AA (88.08)	0.791
		(0.939/0.061)	AG (11.59)	
			GG (0.31)	
**rs11642841**	53845487	C/A	CC (66.77)	0.942
		(0.817/0.183)	CA (29.93)	
			AA (3.29)	

**rs146563001**	53845575	G/A<br>(0.999/0.001)	GG (99.84)<br>GA (0.16)<br>AA (0)	0.984
**rs9972717**	53851304	G/A<br>(0.876/0.124)	GG (76.49)<br>GA (22.26)<br>AA (1.25)	0.515
**rs9925952**	53853898	A/G<br>(0.392/0.608)	AA (15.51)<br>AG (47.33)<br>GG (37.14)	0.863
**rs11075995**	53855291	A/T<br>(0.468/0.532)	AA (16.77)<br>AT (60.03)<br>TT (23.20)	0.000
**rs75929173**	53856104	C/T<br>(0.995/0.005)	CC (99.06)<br>CT (0.04)<br>TT (0)	0.905
**rs74018601**	53857113	C/T<br>(0.969/0.031)	CC (94.04)<br>CT (5.79)<br>TT (0.15)	0.589
**rs10852523**	53858954	T/C<br>(0.632/0.368)	TT (40.13)<br>TC (46.23)<br>CC (13.64)	0.890
**rs61743972**	53860197	G/C<br>(0.411/0.589)	GG (28.68)<br>GC (24.92)<br>CC (46.39)	0.000
**rs3826169**	53860481	G/A<br>(0.269/0.731)	GG (15.83)<br>GA (22.10)<br>AA (62.07)	0.000
**rs10521308**	53865126	G/A<br>(0.969/0.031)	GG (94.04)<br>GA (5.80)	0.589

			AA (0.16)	
**rs10521307**	53865701	A/G	AA (67.24)	0.048
		(0.813/0.187)	AG (28.06)	
			GG (4.70)	
**rs7203572**	53869366	A/C	AA (61.44)	0.000
		(0.710/0.290)	AC (19.12)	
			CC (19.44)	
**rs112728008**	53871621	A/G	AA (96.55)	0.076
		(0.982/0.018)	AG (3.29)	
			GG (0.15)	
**rs17819033**	53873103	G/T	GG (77.11)	0.878
		(0.879/0.121)	GT (21.47)	
			TT (1.41)	
**rs8043785**	53873666	A/G	AA (53.91)	0.022
		(0.722/0.278)	AG (36.52)	
			GG (9.56)	
**rs17219983**	53874918	C/T	CC (51.25)	0.835
		(0.717/0.283)	CT (40.91)	
			TT (7.84)	
**rs12595985**	53876751	C/A	CC (84.17)	0.435
		(0.916/0.084)	CA (14.89)	
			AA (0.94)	
**rs2160481**	53884113	A/G	AA (43.88)	0.428
		(0.668/0.332)	AG (45.76)	
			GG (10.34)	
**rs12448205**	53885683	A/G	AA (40.91)	0.669
		(0.643/0.357)	AG (46.71)	
			GG (12.38)	
**rs9934979**	53886587	C/T	CC (13.63)	0.000

		(0.310/0.690)	CT (34.79)	
			TT (51.56)	
**rs17820328**	53895804	A/G	AA (95.14)	0.332
		(0.975/0.025)	AG (4.70)	
			GG (0.16)	
**rs16952608**	53897352	T/C	TT (92.47)	0.949
		(0.962/0.038)	TC (7.37)	
			CC (0.16)	
**rs28551130**	53897914	T/C	TT (94.67)	0.489
		(0.973/0.027)	TC (5.32)	
			CC (0)	
**rs139397692**	53902297	G/A	GG (98.59)	0.858
		(0.003/0.007)	GA (1.41)	
			AA (0)	
**rs4784329**	53910261	A/C	AA (9.87)	0.864
		(0.312/0.688)	AC (42.63)	
			CC (47.49)	
**rs9934504**	53916879	G/A	GG (83.07)	0.380
		(0.910/0.090)	GA (15.83)	
			AA (1.10)	
**rs56335873**	53920788	T/A	TT (83.07)	0.380
		(0.910/0.090)	TA (15.83)	
			AA (1.10)	
**rs533143696**	53929369	C/T	CC (100)	-
		(100/0)	CT (0)	
			TT (0)	
**rs2111112**	53937632	T/C	TT (4.23)	0.116
		(0.182/0.818)	TC (27.90)	
			CC (67.87)	

**rs9929152**	53939403	G/A (0.231/0.769)	GG (11.91) GA (22.41) AA (65.67)	0.000
**rs79970796**	53939594	G/A (0.996/0.004)	GG (99.21) GA (0.78) AA (0)	0.921
**rs17821532**	53946698	G/A (0.985/0.015)	GG (97.02) GA (2.98) AA (0)	0.703
**rs76299885**	53950231	G/A (0.988/0.012)	GG (97.65) GA (2.35) AA (0)	0.764
**rs12232391**	53953119	T/G (0.472/0.528)	TT (22.88) TG (48.59) GG (28.53)	0.526
**rs16952647**	53954707	G/A (0.981/0.019)	GG (96.39) GA (3.44) AA (0.16)	0.097
**rs16952657**	53956934	C/T (0.933/0.067)	CC (87.46) CT (11.76) TT (0.78)	0.167
**rs77673765**	53965090	G/A (0.992/0.008)	GG (98.43) GA (1.56) AA (0)	0.842
**rs9924072**	53966063	A/G (0.658/0.342)	AA (44.51) AG (42.48) GG (13.01)	0.151
**rs4784333**	53969088	C/G (0.323/0.677)	CC (9.09) CG (46.39)	0.123

			GG (44.52)	
**rs78496669**	53975013	C/T<br>(0.989/0.011)	CC (97.81)<br>CT (2.19)<br>TT (0)	0.779
**rs12933996**	53976662	G/A<br>(0.513/0.487)	GG (26.33)<br>GA (50)<br>AA (23.67)	0.986
**rs72807798**	53978631	C/T<br>(0.992/0.008)	CC (98.59)<br>CT (1.25)<br>TT (0.17)	0.000
**rs16952662**	53978846	G/A<br>(0.988/0.012)	GG (97.65)<br>GA (2.35)<br>AA (0)	0.764
**rs117893287**	53981930	C/T<br>(0.995/0.005)	CC (99.06)<br>CT (0.94)<br>TT (0)	0.905
**rs35090620**	53984623	T/C<br>(0.533/0.467)	TT (28.84)<br>TC (48.90)<br>CC (22.25)	0.655
**rs7192822**	53986221	T/C<br>(0.989/0.011)	TT (97.81)<br>TC (2.19)<br>CC (0)	0.779
**rs35510800**	53988267	G/A<br>(0.713/0.287)	GG (50.78)<br>GA (41.07)<br>AA (8.15)	0.924
**rs16952686**	53989673	A/G<br>(0.966/0.034)	AA (93.42)<br>AG (6.27)<br>GG (0.31)	0.140
**rs1420572**	53993267	C/A	CC (100)	-

		(100/0)	CA (0)	
			AA (0)	
**rs79755695**	53995500	G/A	GG (95.14)	0.529
		(0.976/0.024)	GA (4.86)	
			AA (0)	
**rs17823199**	53998930	C/T	CC (23.82)	0.226
		(0.476/0.524)	CT (47.49)	
			TT (28.68)	
**rs147458024**	54000198	C/T	CC (99.21)	0.921
		(0.996/0.004)	CT (0.78)	
			TT (0)	
**rs1344502**	54000792	G/A	GG (15.20)	0.113
		(0.371/0.629)	GA (43.73)	
			AA (41.07)	
**rs7194907**	54003483	C/T	CC (16.14)	0.136
		(0.384/0.616)	CT (44.51)	
			TT (39.34)	
**rs116290784**	54004229	C/T	CC (98.43)	0.842
		(0.992/0.008)	CT (1.57)	
			TT (0)	
**rs9940629**	54004811	A/G	AA (14.26)	0.000
		(0.222/0.778)	AG (15.83)	
			GG (69.91)	
**rs9932394**	54005099	C/A	CC (16.30)	0.007
		(0.371/0.629)	CA (41.69)	
			AA (42.01)	
**rs76948577**	54006632	C/T	CC (98.59)	0.858
		(0.993/0.007)	CT (1.41)	
			TT (0)	

**rs77422645**	54007640	G/A<br>(0.995/0.005)	GG (99.06)<br>GA (0.94)<br>AA (0)	0.905
**rs113014601**	54008225	A/C<br>(0.987/0.013)	AA (97.49)<br>AC (2.51)<br>CC (0)	0.748
**rs8056299**	54009501	G/A<br>(0.231/0.769)	GG (6.27)<br>GA (33.70)<br>AA (60.03)	0.189
**rs4784335**	54009688	G/T<br>(0.245/0.755)	GG (6.58)<br>GT (35.89)<br>TT (57.52)	0.440
**rs9888758**	54010321	A/G<br>(0.078/0.922)	AA (86.67)<br>AG (10.97)<br>GG (2.36)	0.000
**rs116907816**	54014819	A/C<br>(0.995/0.005)	AA (98.90)<br>AC (1.10)<br>CC (0)	0.889
**rs139054159**	54016869	G/T<br>(0.988/0.012)	GG (97.81)<br>GT (2.03)<br>TT (0.16)	0.002
**rs16952730**	54018921	G/A<br>(0.523/0.477)	GG (31.19)<br>GA (42.16)<br>AA (26.65)	0.000
**rs76536391**	54019653	C/T<br>(0.987/0.013)	CC (97.65)<br>CT (2.04)<br>TT (0.31)	0.000
**rs12324955**	54019686	G/A<br>(0.692/0.308)	GG (49.69)<br>GA (39.02)	0.033

			AA (11.29)	
**rs1125392**	54020145	C/T	CC (8.46)	0.069
		(0.266/0.734)	CT (36.21)	
			TT (55.33)	
**rs8049235**	54021009	G/A	GG (65.83)	0.000
		(0.797/0.203)	GA (27.74)	
			AA (6.43)	
**rs6499662**	54026739	A/G	AA (72.88)	0.008
		(0.846/0.154)	AG (23.35)	
			GG (3.76)	
**rs117071353**	54028708	C/T	CC (99.69)	0.968
		(0.998/0.002)	CT (0.31)	
			TT (0)	
**rs1966435**	54030526	T/C	TT (33.39)	0.010
		(0.556/0.444)	TC (44.36)	
			CC (22.26)	
**rs8046658**	54031563	T/C	TT (23.35)	0.000
		(0.403/0.597)	TC (33.86)	
			CC (42.78)	
**rs11646260**	54032493	A/G	AA (74.61)	0.083
		(0.859/0.141)	AG (22.57)	
			GG (2.82)	
**rs7200972**	54036352	G/A	GG (12.07)	0.007
		(0.313/0.687)	GA (38.40)	
			AA (49.53)	
**rs12931859**	54039486	C/T	CC (36.05)	0.833
		(0.599/0.401)	CT (47.65)	
			TT (16.30)	
**rs9922370**	54040916	A/G	AA (88.24)	0.821

		(0.940/0.060)	AG (11.44)<br>GG (0.31)	
**rs17226942**	54043514	G/A<br>(0.924/0.074)	GG (86.68)<br>GA (11.91)<br>AA (1.41)	0.001
**rs62034069**	54053483	T/C<br>(0.983/0.017)	TT (96.55)<br>TC (3.45)<br>CC (0)	0.658
**rs7194243**	54056159	C/T<br>(0.502/0.498)	CC (27.43)<br>CT (45.45)<br>TT (27.42)	0.022
**rs17825519**	54056753	T/C<br>(0.985/0.015)	TT (97.18)<br>TC (2.66)<br>CC (0.16)	0.020
**rs7195994**	54060205	G/A<br>(0.921/0.079)	GG (84.95)<br>GA (14.26)<br>AA (0.78)	0.586
**rs111977557**	54062556	A/G<br>(0.998/0.002)	AA (99.53)<br>AG (0.47)<br>GG (0)	0.953
**rs12051261**	54065210	C/T<br>(0.706/0.294)	CC (50.94)<br>CT (39.34)<br>TT (9.71)	0.188
**rs62034083**	54068320	A/G<br>(0.984/0.016)	AA (96.71)<br>AG (3.29)<br>GG (0)	0.673
**rs117659448**	54070479	T/C<br>(100/0)	TT (100)<br>TC<br>CC	-

**rs117309839**	54073103	C/T<br>(0.987/0.013)	CC (97.94)<br>CT (2.50)<br>TT (0)	0.748
**rs4784351**	54075698	A/G<br>(0.429/0.571)	AA (25.26)<br>AG (35.27)<br>GG (39.50)	0.000
**rs16952906**	54083167	T/C<br>(0.960/0.040)	TT (92.31)<br>TC (7.37)<br>CC (0.31)	0.312
**rs2540781**	54087859	C/A<br>(0.894/0.106)	CC (81.97)<br>CA (14.89)<br>AA (3.13)	0.000
**rs856973**	54093181	G/T<br>(0.947/0.053)	GG (89.50)<br>GT (10.34)<br>TT (0.16)	0.524
**rs117849391**	54097226	C/A<br>(0.997/0.003)	CC (99.37)<br>CA (0.63)<br>AA (0)	0.937
**rs12446690**	54098681	C/A<br>(0.946/0.054)	CC (89.50)<br>CA (10.19)<br>AA (0.31)	0.917
**rs2003583**	54100006	C/T<br>(0.591/0.409)	CC (37.93)<br>CT (42.32)<br>TT (19.75)	0.002
**rs62034115**	54105336	T/C<br>(0.981/0.019)	TT (96.24)<br>TC (3.76)<br>CC (0)	0.628
**rs9302656**	54108991	G/A<br>(0.942/0.058)	GG (88.87)<br>GA (10.66)	0.536

			AA (0.47)	
**rs75361495**	54109412	A/G<br>(100/0)	AA (100)<br>AG (0)<br>GG (0)	-
**rs7206012**	54113564	T/C<br>(0.964/0.036)	TT (92.79)<br>TC (7.21)<br>CC (0)	0.345
**rs967515**	54114217	C/T<br>(0.951/0.049)	CC (90.28)<br>CT (9.72)<br>TT (0)	0.197
**rs59134332**	54114242	A/G<br>(0.993/0.007)	AA (98.59)<br>AG (1.41)<br>GG (0)	0.858
**rs16953002**	54114824	G/A<br>(0.929/0.071)	GG (87.93)<br>GA (10.03)<br>AA (2.04)	0.000
**rs708258**	54115369	A/G<br>(0.687/0.313)	AA (49.69)<br>AG (38.09)<br>GG (12.23)	0.004
**rs1008400**	54119892	T/C<br>(0.618/0.382)	TT (38.87)<br>TC (45.92)<br>CC (15.20)	0.495
**rs11646512**	54120952	A/G<br>(0.621/0.379)	AA (38.71)<br>AG (46.87)<br>GG (14.42)	0.921
**rs78638652**	54122162	C/T<br>(0.992/0.008)	CC (98.43)<br>CT (1.57)<br>TT (0)	0.842
**rs11863548**	54123512	G/A	GG (83.39)	0.000

		(0.895/0.105)	GA (12.23)<br>AA (4.39)	
**rs708250**	54125040	G/A<br>(0.984/0.020)	GG (0.16)<br>GA (3.61)<br>AA (96.24)	0.120
**rs2665271**	54127879	T/C<br>(0.585/0.415)	TT (17.71)<br>TC (47.65)<br>CC (34.64)	0.633
**rs62034138**	54129948	G/A<br>(0.841/0.159)	GG (71.32)<br>GA (25.55)<br>AA (3.13)	0.254
**rs71392011**	54150035	C/A<br>(0.986/0.014)	CC (97.18)<br>CA (2.82)<br>AA (0)	0.718
**rs2542681**	54150216	A/T<br>(0.204/0.796)	AA (5.17)<br>AT (30.41)<br>TT (64.42)	0.112
**rs191211628**	54151267	T/C<br>(0.989/0.011)	TT (97.81)<br>TC (2.19)<br>CC (0)	0.779
**rs12597001**	54151411	G/T<br>(0.643/0.357)	GG (45.61)<br>GT (37.30)<br>TT (17.08)	0.000
**rs12444954**	54155047	A/G<br>(0.551/0.449)	AA (46.08)<br>AG (18.03)<br>GG (35.89)	0.000
**rs531076397**	54155348	G/T<br>(100/0)	GG (100)<br>GT (0)<br>TT (0)	-

**rs11865975**	54155409	C/T<br>(0.994/0.006)	CC (98.75)<br>CT (1.25)<br>TT (0)	0
**rs77601508**	54155511	G/T<br>(0.999/0.001)	GG (99.84)<br>GT (0.16)<br>TT (0)	0.984
**rs12929934**	54155686	G/A<br>(0.733/0.267)	GG (57.37)<br>GA (31.82)<br>AA (10.82)	0.000
